# Management of ventricular septal defect with pulmonary atresia and major aorto pulmonary collateral arteries: Challenges and controversies

**DOI:** 10.4103/0974-2069.74040

**Published:** 2010

**Authors:** KS Murthy, K Pramod Reddy, R Nagarajan, V Goutami, KM Cherian

**Affiliations:** Madras Medical Mission, Chennai and Innova Children’s Heart Hospital, Hyderabad, India

## INTRODUCTION

Tetrology of Fallot (TOF) comprises 3.9% of congenital heart disease,[1] of which approximately 5–10% have pulmonary atresia (PA) with ventricular septal defect (VSD). Two-thirds of the cases with PA are associated with major aorto pulmonary collateral arteries (MAPCAs).[2] Survival rate without surgery can be as low as 50% at 1 year of age and 8% at 10 years.[3] TOF, PA with MAPCAs is a complex congenital cardiac anomaly and one of the most challenging groups to manage surgically. Earlier, these patients were treated with multistage unifocalization of MAPCAs through thoracotomies, followed by final repair via median sternotomy.[4–6] An aggressive approach involving single-stage complete unifocalization and complete repair through median sternotomy is described by Reddy *et al*.[7] and by us.[8] The surgical treatment for this malformation is evolving and no standard protocols have been described.

In this article, we present our surgical experience (both from Madras Medical Mission, Chennai, and Innova Children’s Heart Hospital, Hyderabad) with single-stage complete unifocalization and repair, and discuss the challenges and controversies associated with the management of this difficult subset.

## EMBRYOLOGICAL ASPECTS

Variation of pulmonary blood supply is the most distinctive feature of this anomaly. This is because the lungs develop from the foregut, and their nutrient supply, as that of the esophagus, arises initially from the dorsal aortic plexus.[9] However, at about day 27 in the antenatal period, arterial branches of the paired 6th aortic arch form an anastomosis with the pulmonary vascular plexus. As a result, the lung receives dual supply. With time, the branches from the 6th arch enlarge and those from the descending aorta become comparatively smaller. Persistence of the branches from the aorta in postnatal life forms the MAPCAs. They are variable in their origin, number, size, course, and arborization. The natural history of the MAPCAs follows a course of progressive stenosis and occlusion. Thus, the sooner these collateral arteries are unifocalized and normal physiologic circulation is established, the greater is the number of healthier lung segments that can be incorporated into the pulmonary circulation. The native pulmonary arteries may be present or absent. If they are present, they are either normal sized or hypoplastic, and confluent or nonconfluent. An area of the lung may receive its blood supply from the native pulmonary arteries or MAPCAs either singly or in combination. Depending on these variations, these patients are initially seen with either cyanosis, caused by insufficient pulmonary blood flow, or congestive heart failure or pulmonary hypertension, caused by excessive pulmonary flow. A few patients may have a balanced pulmonary blood flow that permits them to survive to adulthood.

## DIAGNOSIS

The initial diagnosis is made by means of echocardiography and confirmed with the help of cardiac catheterization and angiocardiography. An effort is made to obtain pressures in individual MAPCAs. Selective angiographic delineation of MAPCAs is obtained in the ascending aorta, arch, descending aorta, and brachiocephalic arteries on either side. It is important to delineate the origin of each MAPCA and its course preoperatively in order to isolate each and plan the surgical options.

Three-dimensional (3D) imaging technology used in procedures such as magnetic resonance imaging (MRI), helical computed tomography (CT) and electron beam CT has progressed signinificantly, allowing 3D visualization of pulmonary arteries and MAPCAs which is crucial for preoperative planning.[10]

## CLINICAL SPECTRUM

Between June 1997 and April 2010, 124 patients with TOF or its variants with MAPCAs underwent single-stage complete unifocalization, with or without final repair. Their age ranged from 3 months to 24 years (median 3.2 years), and weight ranged from 4 to 50 kg (median 15 kg).

## MORPHOLOGY

**Figure 1:**
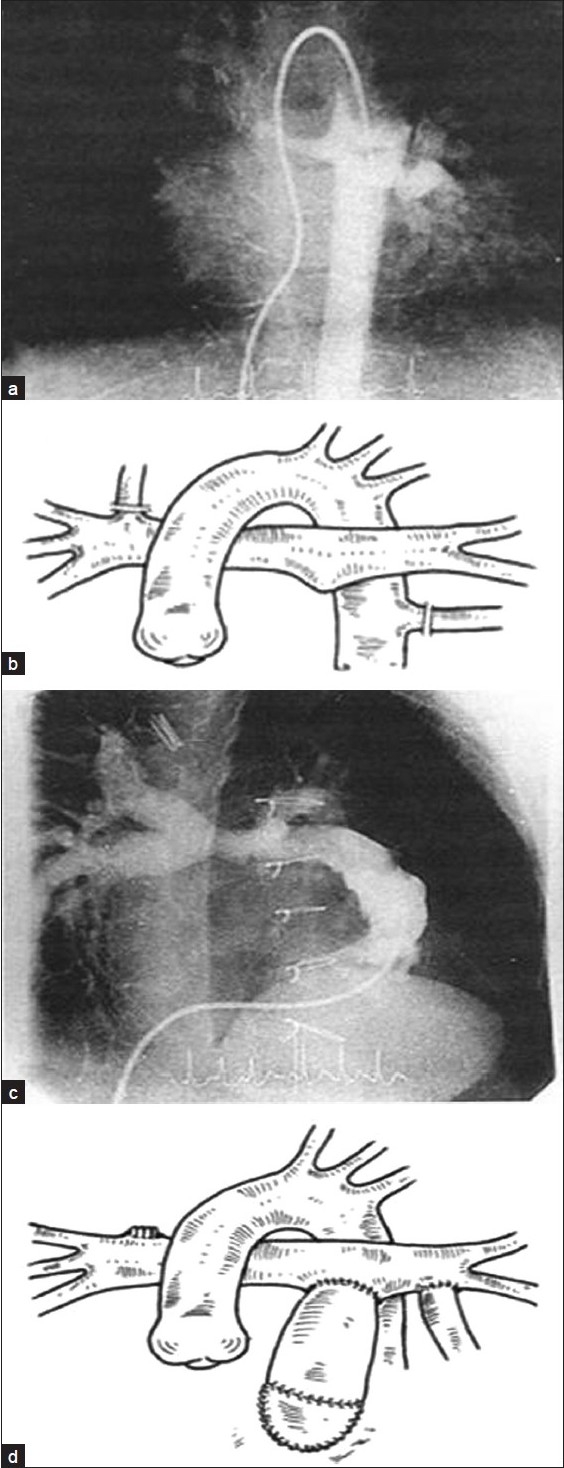
Well formed native pulmonary arteries with MAPCAs (group I). (a) Preoperative angiography; (b) preoperative diagram; (c) postoperative angiography; (d) postoperative diagram

**Figure 2:**
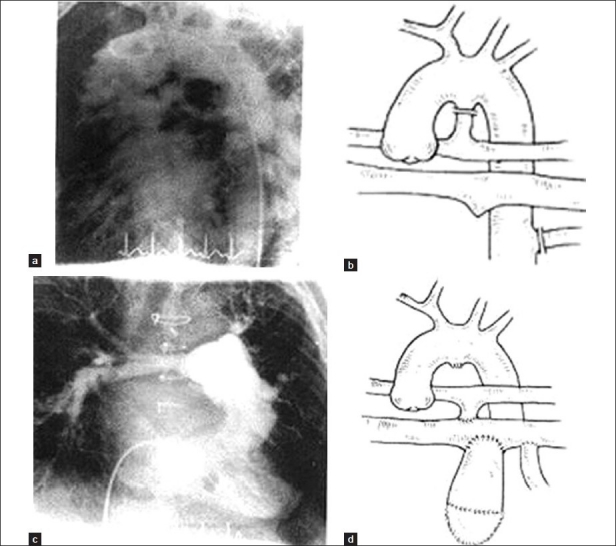
Hypoplastic PAs and MAPCAs (group II). (a) Preoperative angiography; (b) preoperative diagram; (c) postoperative angiography; (d) postoperative diagram

**Figure 3:**
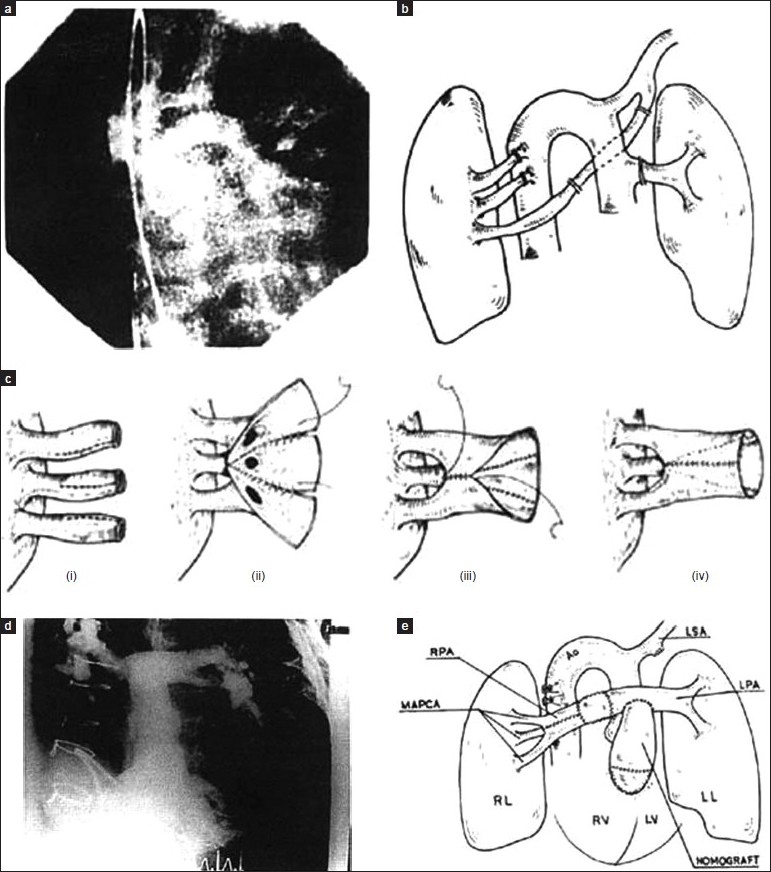
Only MAPCAs (no active PAs group III). (a) Preoperative angiography; (b) preoperative diagram; (c) reconstruction of neopulmonary arteries; (d) postoperative angiography; (e) postoperative diagram

We identified three groups according to the morphologic features of the pulmonary arteries and MAPCAs and their arborization pattern in the lungs. Group I had well-formed native pulmonary arteries with MAPCAs, group II had hypoplastic pulmonary arteries with MAPCAs, and group III had only MAPCAs without native pulmonary arteries. The three groups were further subcategorized into those patients with protected MAPCAs with proximal stenosis, and those with unprotected MAPCAs (unobstructed flow) [Figures 1–3]. A total of 386 MAPCAs were unifocalized. The details of origin of the MAPCAs are shown in Table 1.

**Table 1:** Profile of MAPCAS

Total no. of MAPCAs	386
Range	1–5 (mean 3)
Descending aorta	266(69)
Subclavian	82(21)
Arch	30(8)
Ascending aorta	8(2)

MAPCAs, major aorto pulmonary collateral arteries

## GOALS OF OPERTIVE THERAPY

Our aim was to establish a single source of blood supply to the lungs from the pulmonary arteries and MAPCAs, achieve tissue-to-tissue anastomosis, and avoid or minimize the use of prosthetic material in order to promote future growth in the children. Even though there might be dual supply to the lung if the MAPCAs were more than 2 mm in size, they were still unifocalized in order to maximize the pulmonary arterial bed available for final correction, on the assumption that the postoperative right ventricle (RV) pressure would be lower.[11]

## TYPES OF SURGICAL PROCEDURE

All the patients had single-stage complete unifocalization. According to the availability of size and number of pulmonary vascular segments, they had one of the following three options of surgical treatment: (1) final repair (closure of the VSD and RV-to-pulmonary artery conduit), (2) RV-to-pulmonary artery conduit alone (VSD left open), or (3) central shunt from ascending aorta to reconstructed new pulmonary artery with a polytetrafluoroethylene (PTFE) graft. If the size of the pulmonary arteries and MAPCAs was more than 75% of expected or more than 15 segments of the lung were unifocalized, final repair was done. If the size of the pulmonary arteries and MAPCAs was between 50 and 75% of normal or the pulmonary segments were between 10 and 14, an RV-to-pulmonary artery conduit was employed without closure of the VSD. If the size of the pulmonary arteries was less than 50% of expected or there were less than 10 segments which could be unifocalized, only a central shunt was performed.[11]

## INDICATIONS FOR OPERATION

The surgical management of this complex anomaly should be individualized according to (1) arborization of pulmonary vasculature, (2) amount of pulmonary blood flow, (3) morphology and sizes of the native pulmonary arteries and MAPCAs, and (4) the age of the patient. The younger the patient, the better is the prognosis. In the protected pulmonary vasculature (MAPCAs with proximal stenosis), final repair or an RV-to-pulmonary artery conduit or a central shunt should be done according to total availability of the pulmonary segments and size of the central pulmonary arteries. In infants less than 1 year of age with hypoplastic or absent central pulmonary arteries with unprotected MAPCAs (before they develop irreversible pulmonary vascular disease) or those with protected MAPCAs with proximal stenosis, any one of the three surgical options can be offered according to the total size of the reconstructed new pulmonary arteries. In patients with hypoplastic or absent pulmonar arteries with unprotected MAPCAs, who are older than 1 year, all surgical options have a questionable outcome.[11]

## SURGICAL TECHNIQUES

### Median sternotomy

**Figure 4:**
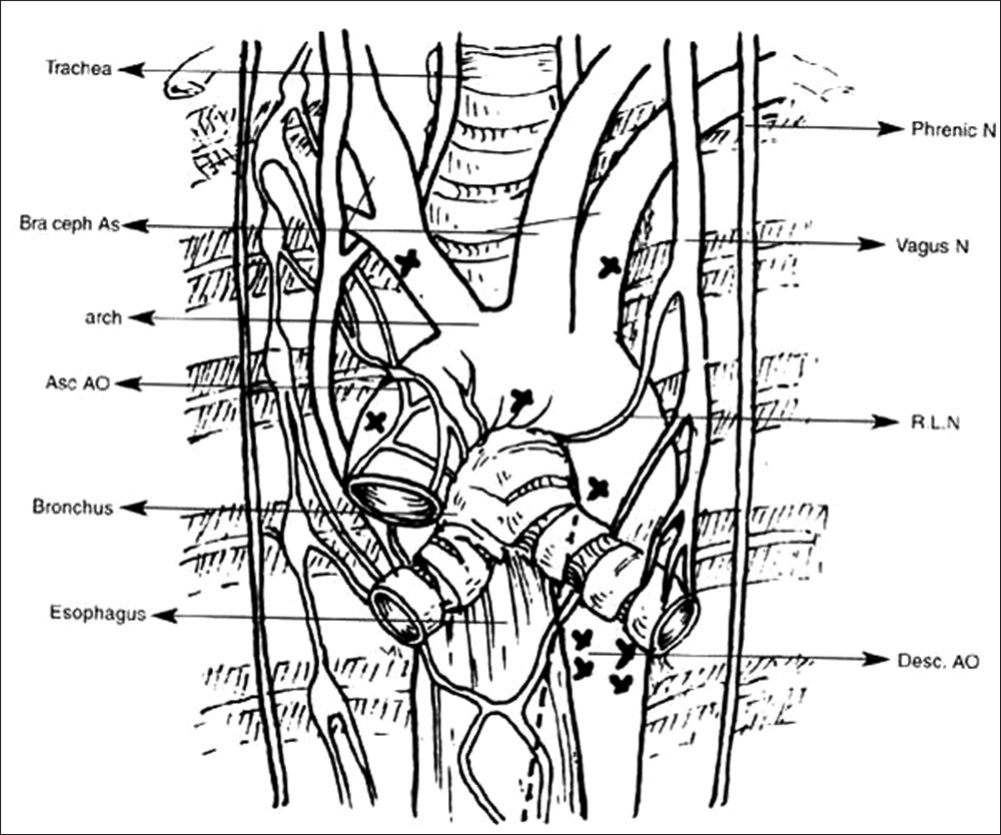
Important mediastinal structures (X mark showing the site of origin of MAPCAs). MAPCAs, major aorto pulmonary collateral arteries; N, nerve; RLN, recurrent laryngeal nerve; ASC Ao, ascending aorta; Desc Ao, descending aorta; Bra ceph As, brachiocephalic arteries

After routine median sternotomy, pericardium was harvested and treated with 0.6% gluteraldehyde for 20 minutes for subsequent use. The native pulmonary arteries (if present) were dissected and isolated to their hilar region. The ascending aorta and the superior vena cava (SVC) were freed from surrounding tissues in order to retract them freely to get satisfactory access to the deeper plane for the dissection. The MAPCAs were approached by dissecting along the aorta and the brachiocephalic arteries, as per their course delineated by preoperative angiography, to a sufficient length from their origin to bring them to the transverse sinus without any tension during anastomosis. Opening of the pleura was not necessary because of limited dissection. The descending aortic MAPCAs were reached by dissecting in the posterior mediastinum after opening the posterior pericardium. In patients with left aortic arch, the descending aortic MAPCAs were approached by dissecting between the area of the left side of the ascending aorta and the left atrium, and above or below the left main bronchus. While dissecting on the left side, care was taken not to compress the left coronary artery and its branches during retraction. In patients with right aortic arch, the descending aortic MAPCAs were reached by approaching between the ascending aorta, SVC, and the roof of the left atrium, usually above or below the carina and the right main bronchus. During dissection, care was taken to avoid hemodynamic compromise by momentarily stopping the dissection and retraction. Precautions were taken not to injure the trachea, bronchi, esophagus and phrenic, vagus, and recurrent laryngeal nerves [Figure 4]. All the MAPCAs were looped before initiating cardiopulmonary bypass (CPB). Failure to do so creates difficulties in initiation and maintenance of adequate perfusion pressures during CPB as the large collaterals steal from the systemic perfusion. In addition, the duration of CPB can be minimized using this approach.

Under CPB and beating heart at normothermia, all the MAPCAs were disconnected from their origin and their aortic ends were closed. They were then anastomosed end-to-side or side-to-side to native pulmonary arteries (if present); otherwise, MAPCA-to-MAPCA [Figures 1–3] anastomosis was performed using continuous 8-0 polypropylene sutures. Tissue-to-tissue anastomosis was preferred to allow for future growth in these children.

Following these procedures, the aorta was cross clamped and the heart was arrested by administering cardioplegia solution. Under cardioplegia arrest, the VSD was closed with a PTFE patch (trans RV), and RV to pulmonary artery continuity was established with a cryopreserved aortic or pulmonary homograft conduit. If the resultant MAPCA-PA diameter was not suitable for complete repair, the VSD was left open or a central shunt was performed from the ascending aorta to the pulmonary arteryPA with a PTFE interposition graft on a beating heart.

### Clamshell approach

**Figure 5:**
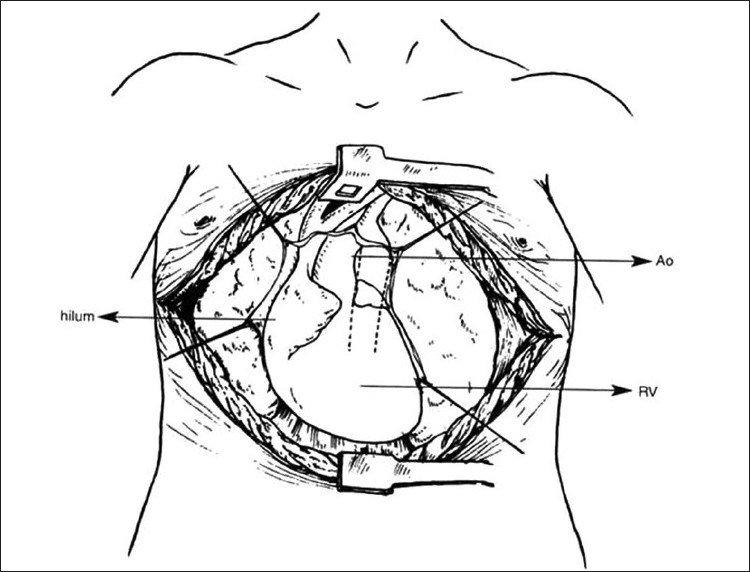
Clamshell approach. Ao, Aorta; RV, right ventricles

The clamshell approach was preferred in patients who have undergone a previous surgical intervention such as median sternotomy for a central shunt or an attempted surgical repair. Previous surgical intervention results in extensive scarring of tissues around the mediastinum and the hilar region. In this approach, there was no need for extensive dissection. It gave excellent exposure to both hilar regions, the heart, and the great vessels. In this approach, a submammary skin incision was made, extending laterally to the anterior axillary line and both the pleural cavities were entered through the fourth intercostal space. The internal mammary artery and vein were ligated and divided on either side. The sternum was divided transversely. The remainder of the dissection and subsequent procedure were similar to that of the median sternotomy approach [Figure 5].

## RESULTS

### Early results

In our experience with 124 patients between 1997 and 2010, median sternotomy approach was used in 116 patients, and clamshell approach was used in 8 patients. All the patients had a single-stage complete unifocalization. The postoperative results are shown in Table 2.

**Table 2:** Results

Group	Complete unifocalization	Final correction	RV-PA conduit	Central shunt	Mortality
Total (n=124)	All	74(60%)	26(21%)	24(19%)	16(13%)
Group I (*n*=42)	All	42	–	–	Nil
Group II (*n*=40)	All	17	12	11	8
Group III (*n*=42)	All	15	14	13	8

Figures in parenthesis are in percentage

These 124 patients had 148 procedures (1.2 procedures per patient). All the patients had a single-stage complete unifocalization, of whom 60% (*n*=74) had final repair (VSD closure and RV-to-pulmonary artery conduit), 21% (*n*=26) had an RV-to-pulmonary artery conduit alone (VSD left open), and 19% (*n*=24) had a central shunt. Tissue-to-tissue anastomosis was achieved in most of the patients In 10 patients, homograft tissue was used for augmentation, and in one patient, a 14-mm PTFE tube graft was used to achieve the confluence of the pulmonary arteries. The mean CPB time was 148±29 minutes, and the mean aortic cross clamp time was 45±21 minutes. In the final repair group, the mean ratio of RV to left ventricular (LV) pressure was 0.66 (range 0.38-0.9), and the systemic oxygen saturation ranged from 95 to 100%. In the patient in whom the VSD was left open and an RV-to-pulmonary artery conduit or a central shunt was done, the systemic saturation ranged from 85 to 96% (mean 92%) and from 72 to 88% (mean 78%), respectively.

### Mortality and morbidity

**Figure 6:**
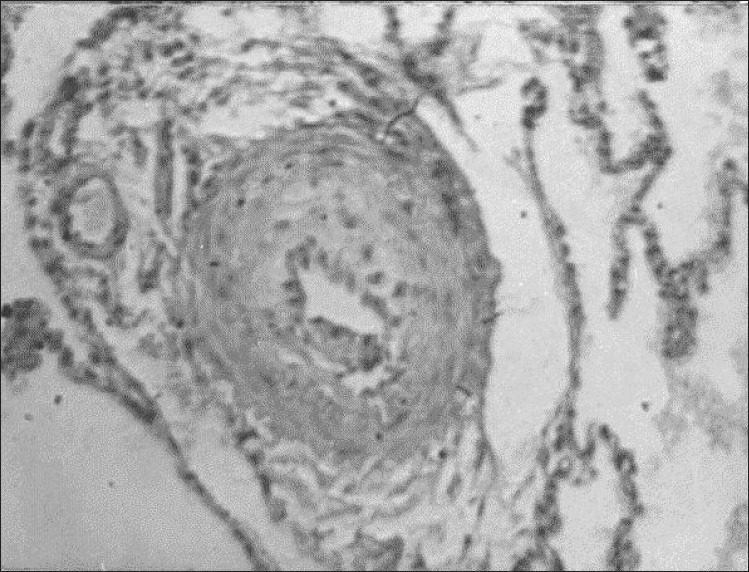
Grade III (Heath-Edwards) pulmonary vascular changes

There were 16 early deaths (13%). There were no deaths in group I and eight deaths each in groups II and III. The first patient had complete unifocalization and final repair and could not come off bypass because he had suprasystemic RV pressure. He was placed back on bypass and the VSD patch was removed. He had prolonged bypass time, developed RV dysfunction and died on the seventh postoperative day. The second death was of a 1-year-old child who had a complex unifocalization and an RV-to-pulmonary artery conduit without VSD closure. His saturation was maintained between 85 and 92% with an FIO_2_ (fraction of inspired oxygen) of 0.3. A low cardiac output gradually developed, and he died on the second postoperative day. At autopsy, a 4-mm MAPCA in the lower part of the descending aorta was noted, which was missed on preoperative angiography. The third patient was a 7-year-old boy with unprotected MAPCAs without native pulmonary arteries, who had complete unifocalization and an RV-to-pulmonary artery conduit. Gradual desaturation and ventricular failure developed, and he died on the fourth postoperative day. At autopsy, the lung specimen showed grade III pulmonary vascular changes according to the Heath-Edwards classification [Figure 6]. The fourth patient died of intractable bleeding on the first postoperative day. Other patients died of low cardiac output and multiorgan failure. Four patients had re-exploration for excessive mediastinal bleeding. Two patients had unilateral phrenic palsy, and one patient had bilateral phrenic palsy and required prolonged ventilation.

### Follow up

The follow up ranged from 1 month to 12 years. All the patients had echocardiography to asses RV function, conduit position and distal pulmonary artery diameter at the time of discharge and during follow up.

The first 16 patients had cardiac catheterization and angiocardiography, either before they were discharged from the hospital or after 3 months of follow up, to assess the status of the repair. The patients who had an RV-to-pulmonary artery conduit without VSD closure had cardiac catheterization and angiocardiography performed within 3–12 months of the initial operation. Indications for completion of VSD closure were congestive heart failure requiring decongestive therapy, improved resting oxygen saturation by pulse oxymeter, and a predominant left-to-right shunt demonstrated by echocardiography. These findings were confirmed by cardiac catheterization. If the left-to-right shunt was greater than 2:1 with an increased shunt fraction on 100% FIO_2_, the VSD was closed.

There were 16 reoperations. Fifteen patients had successful completion of VSD closure and one patient had reconstruction of pulmonary artery stenosis (a PTFE graft was used to achieve the pulmonary confluence). There were three late deaths. Two of these patients developed a progressive increase in RV pressure and died 2 and 2.5 years, respectively, after the initial operation. Endocarditis developed in one patient who died 2 months after surgery. Balloon dilatation of stenosed MAPCAs was done in three patients (one MAPCA in each patient).

## DISCUSSION

Patients with TOF, PA, and MAPCAs remain one of the most challenging groups to manage surgically. Untreated, most patients die early in life, either with severe cyanosis and its attendant complications or with congestive heart failure and progressive pulmonary vascular disease. Earlier, these patients were treated with conventional multistage procedures requiring an average of three procedures (range 2–6) before complete repair. This culminated in final repair in 11.5–60.5% of patients. Overall mortality while achieving complete repair ranged from 10.2 to 19.2%.[4–6] From 1993 to May 1997, these patients were treated with multistage unifocalization in our institute. Fourteen patients had 21 procedures (1.5 procedures per patient), in which only three of them had complete repair. There were two deaths (14%).[8]

The microvasculature of the lungs in patients with MAPCAs is healthier at birth. The natural history of MAPCAs often follows a course of progressive stenosis and occlusion, sometimes precluding access to a given segment of the lung. Long standing severe stenosis may lead to distal arterial hypoplasia and underdevelopment of preacinar and acinar vessels and alveoli.[12] In staged unifocalization, the use of nonviable conduits with a tendency to occlusion may lead to a loss of the lung segments. Infancy is characterized by the highest rate of attrition, and without surgery, almost 50% of the patients die before 1 year of age. Any surgical procedure designed to have an impact on the natural history of these patients must alter the pattern of attrition in infancy. If delayed staged unifocalization is performed, only 20–30% of a cohort of newborns will achieve final repair. Most staged approaches are based on the concept of an initial phase to increase the native pulmonary artery blood flow, in an effort to stimulate growth. Various strategies have served to advance the field and have provided some good results in a select group of patients, but many patients remain without final repair. When the staged approach is used, it is difficult to achieve final repair in patients with nonconfluent, hypoplastic, and absent native pulmonary arteries.[5] Hypoplastic central pulmonary arteries was a significant risk factor in this disease. By using a strategy of unifocalization, intrapericardial pulmonary artery reconstruction, and RV–pulmonary artery conduit, excellent long-term survival can be achieved in this group of patients even in the absence of native intrapericardial pulmonary arteries.[13,14] For this reason, an aggressive approach consisting of single-stage unifocalization and repair has been started.[7,8] From June 1997 onward, we began performing single-stage unifocalization, and 124 patients have been treated with this technique. There was no mortality among patients in group I (those with well-formed pulmonary arteries and MAPCAs). There were eight deaths, each in groups II and III, indicating that these two groups are the difficult spectrum regarding decision making, surgical technique, and development of progressive pulmonary vascular disease.

Sixty percent of the patients had final repair, 21% of the patients had an RV-to-pulmonary artery conduit, and 19% of the patients had a central shunt. Fifteen patients had completion of VSD closure. In the McElhinney group,[15] 64% of the patients had final repair. Their age ranged from 2 weeks to 37 years (mean 7.3 months), and 65% were younger than 1 year. In that study, the follow up ranged from 1 to 61 months. There were 6 late deaths, and 14 patients underwent completion of VSD closure. In our experience, the follow up ranged from 1 month to 12 years. There were 3 late deaths, and 15 patients underwent completion of VSD closure as a final repair. In both the experiences, the primary aim is to achieve tissue-to-tissue anastomosis for future growth. Both the groups used calcium-supplemented warm blood prime to maintain the beating heart during the unifocalization procedure.

There were some differences between our approach and that of Hanley’s group. We used the routine median sternotomy incision in usual cases and the clamshell approach in previous sternotomy patients for unifocalization. We dissect the MAPCAs from their origin to a sufficient length to reach for the anastomosis. The dissection is limited, and injury to the phrenic nerve and other important structures may be less. Opening of the pleura is rarely necessary to identify the MAPCAs for dissection.[11] In Hanley’s technique, a generous midline median sternotomy incision is used. Both the pleural spaces are opened widely anterior to the phrenic nerves and the lungs are lifted out of the pleural cavities, allowing identification of the collaterals at their aortic origins.[7] We choose the type of surgery, such as final repair, an RV-to-pulmonary artery homograft, or a central shunt, according to the total size of the pulmonary arteries and MAPCAs and their segmental distribution, as shown by angiocardiography and intraoperative assessment. In Hanley’s technique, intraoperative pump flow is used to check the pulmonary artery pressure and accordingly decide the type of surgery. If arborization is inadequate, it would be better not to close the VSD.[16] Even though there is a correlation between the total neopulmonary artery index (TNPAI; Nakata index)[17] and the postoperative RV/LV pressure ratio, there was a substantial overlap in the TNPAI below 200 mm^2^/m^2^.[17] It is difficult to estimate the Nakata index because of arborizational abnormalities, technical problems in reconstruction of neopulmonary arteries, and distal MAPCA stenosis.

## CONTROVERSIES

Patients with unobstructed MAPCAs have the same clinical course as do other patients with aortopulmonary shunts, such as those who have truncus arteriosus or aortopulmonary window. In patients with unobstructed MAPCAs, we suggest surgical intervention before the age of 1 year, before irreversible pulmonary vascular changes or prior to development of stenosis in MAPCAs.[11,17] Two patients in our experience died with suprasystemic RV pressures at 2 and 2.5 years after final repair. They had a progressive increase in RV pressure even though the immediate postoperative PRV/LV ratios were 0.7 and 0.4, respectively. We presume that these patients had progressive pulmonary vascular disease. Based on this experience, we began fenestration of the VSD patches in final repair patients.

**Figure 7:**
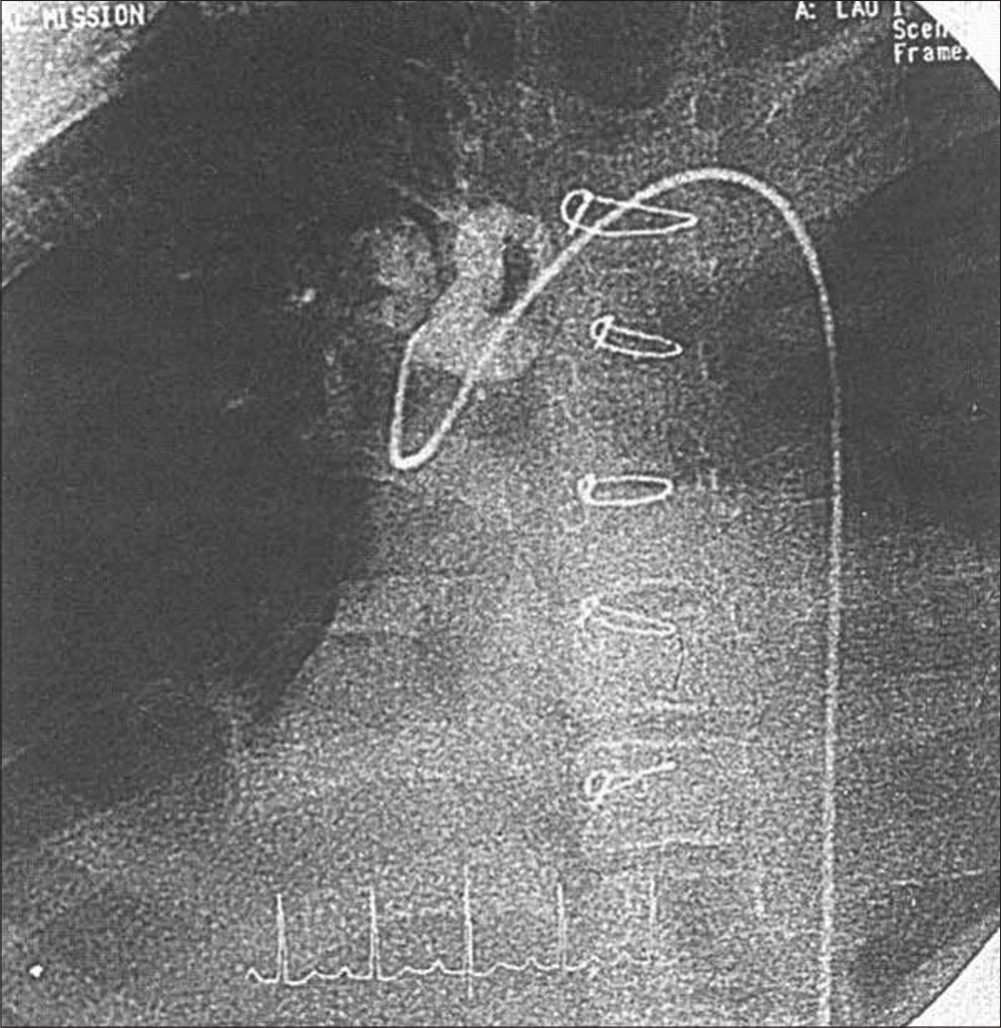
Angiography of unifocalization and central shunt

**Figure 8:**
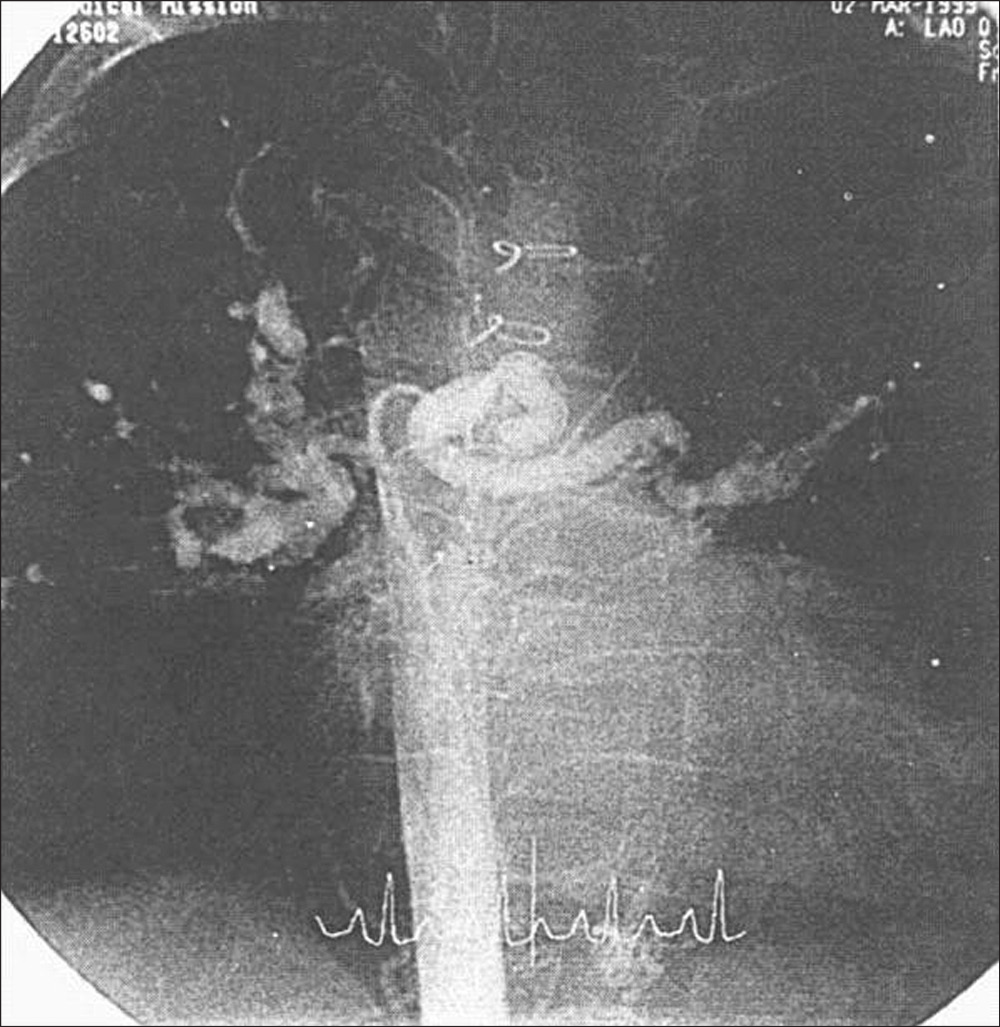
Angiography of MAPCAs with multiple distal stenosis. MAPCAs: major aortopulmonary collateral arteries

In our experience, the patients who had a central shunt (pulmonary vascular segments < 10) have not shown much improvement in pulmonary vascular growth and did not come for final repair or at least for RV-to-pulmonary artery conduit [Figure 7]. It is doubtful whether surgical intervention in these patients changes the natural history of TOF, PA, and MAPCAs. It is difficult to repair all multiple distal stenosis of the MAPCAs [Figure 8]. Other extreme forms occur in which there are no demonstrable native pulmonary arteries or MAPCAs by angiocardiography. Instead, multiple small collaterals are present, which supply both the lungs. These patients present with severe cyanosis. We feel that in these patients also, we are not able to offer any surgical intervention to improve their condition.

## CONCLUSION

With single-stage unifocalization, patients are able to undergo complete repair, preferably at an early age. Complete repair achieves early normalization of cardiovascular physiology with correction of cyanosis or pulmonary hypertension and attendant complications. Early single-stage unifocalization reduces the number of operations and hospitalizations, and thereby, is less expensive than a multistage procedure. By achieving tissue-to-tissue anastomosis, we can expect future growth in these children. Approaching the MAPCAs through a median sternotomy or a clamshell incision is safe and reproducible. Early and midterm results are encouraging, and ongoing follow-up allows identification of those patients who will benefit from surgery.
